# Mineral lick use by a community of large herbivores in northern Iran

**DOI:** 10.1002/ece3.9731

**Published:** 2023-01-18

**Authors:** Farid Salmanpour, Zahra Shakoori, Mehdi Kia, Rahman Eshaghi, Mehdi Ghaderi, Saied Ghomi, Reza Kaveh, Kuros Rabie, Bahram H. Kiabi, Mohammad S. Farhadinia

**Affiliations:** ^1^ Department of Biodiversity and Ecosystem Management, Research Institute of Environmental Sciences Shahid Beheshti University Tehran Iran; ^2^ Department of Plant Science and Biotechnology, Faculty of Science and Biotechnology Shahid Beheshti University Tehran Iran; ^3^ Department of Environment Mazandaran Provincial Office Mazandaran Iran; ^4^ Department of Animal Sciences and Marine Biology, Faculty of Life Sciences and Biotechnology Shahid Beheshti University Tehran Iran; ^5^ Department of Biology University of Oxford Oxford UK; ^6^ Durrell Institute of Conservation and Ecology, School of Anthropology and Conservation University of Kent Kent UK

**Keywords:** Caspian red deer, geophagy, Iran, lick site, roe deer

## Abstract

Natural mineral licks are ecologically valuable resources to meet the physiological needs of herbivores, particularly in temperate forests. Importantly, licking sites can harbor high anthropogenic risk for conservation‐dependent herbivores through higher chance of pathogen spillover from livestock and increased levels of poaching risks. However, to the best of our knowledge, there is no information on the mineral lick use in temperate forests of west Asia and the Caucasus where a few threatened deer species exist. We monitored four naturally occurring mineral licks in Central Alborz Protected Area, northern Iran during May–July 2019 using camera traps and analyzed the mineral content of the licking sites. A total of 53 independent mineral lick visits were obtained from only three species of herbivores, i.e., Caspian red deer (*Cervus elaphus maral*; *n* = 21), roe deer (*Capreolus capreolus*; *n* = 26), and wild pig (*Sus scrofa*; *n* = 6). The sex ratio of visiting Caspian red deer was highly skewed toward females (3M:18F), whereas it was more balanced in visiting roe deer (11M:15F). The species‐level distribution of visits corresponded to diurnal and cathemeral for Caspian red deer and for roe deer, respectively, without any evidence of different activity curves. There was a negative nonlinear relationship between the ambient temperature and the visitation rate. Our findings showed that mineral licks are important habitat features for these large herbivores and need to be included in spatial mapping and habitat protection measures.

## Graphical abstract

Natural mineral licks are ecologically valuable resources to meet the physiological needs of herbivores, particularly in temperate forests. Importantly, licking sites tend to have higher anthropogenic risk on conservation‐dependent herbivores through higher chance of pathogen transmission from livestock and higher levels of poaching risks. However, to the best of our knowledge, there is no information on the mineral lick use in temperate forests of west Asia and the Caucasus where a few threatened deer species exist.

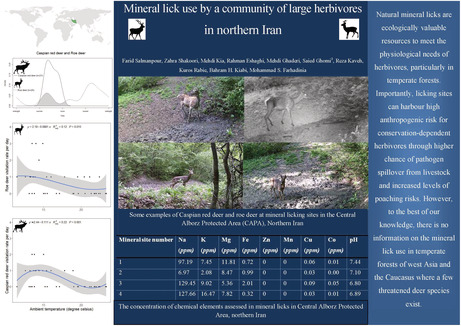

## 1 INTRODUCTION

Understanding the species dietary requirements as a component of wildlife ecology and management is important for predicting the individual and population fitness (Griffiths et al., 2020). As a vital part of the species diet, minerals form a diverse group of nutrients that make up only 5% of the body's composition, but they play an essential role in the body's function (Matsubayashi et al., 2006). Minerals required by living organisms include major elements, such as calcium, phosphorus, potassium, sodium, magnesium, and trace elements such as iron, zinc, manganese, copper, molybdenum, iodine, selenium, and cobalt (Atwood & Weeks, 2003).

Sodium is one of the most important mineral elements in the physiological performance of animals (Atwood & Weeks, 2003). Sodium is demanded for regulating body fluids, contracting muscles, and transmitting messages to the nervous system (Kaspari, 2020), particularly in high‐demand stages, such as lactation in females (Ceacero et al., 2009) and antlerogenesis in males (Atwood & Weeks, 2003). Sodium is usually present in low concentrations in plants, so many herbivores may be deficient in this element (Kaspari, 2020). Wherever sodium and other minerals are deficient in the herbivores' diet, licking minerals will supplement their needs, a behavior commonly known as geophagy (Griffiths et al., 2020).

Mineral licking sites are natural sites that are generally composed of erosive geological materials (Ceacero et al., 2009). Their utilization is associated with the chemical composition as well as the surrounding habitat type and vegetation (Razali et al., 2020). There are also interspecific and seasonal variation in species use of mineral sites (Blake et al., 2011; Tobler et al., 2009). Interspecific behavioral differences at mineral licks, such as the visitation rate and the temporal patterns, can determine the extent to which species need minerals (Blake et al., 2011; Griffiths et al., 2020). Nonetheless, they have never been studied in Hyrcanian forests of northern Iran, where a few large herbivores such as Caspian red deer (*Cervus elaphus maral*), roe deer (*Capreolus capreolus*) and wild pig (*Sus scrofa*) exist. These sites are also crucial for the conservation of the herbivores, as they are widely known among local poachers to be frequently visited by the species, so can create a poaching hotspot (Blake et al., 2011), particularly for the Caspian red deer which suffers from a drastic population reduction due to poaching and habitat loss across its range in west Asia (Shokri et al., 2021).

In this study, our objectives were twofold: (1) understanding which species of herbivores visit mineral licks in this area and (2) identifying factors associated with herbivore activities at mineral licks, including potential intraspecific competition, average daily temperatures, and mineral lick nutrient concentrations. Eventually, we highlighted the vulnerability of the licking sites to local poachers which are occasionally active in the area.

## 2 MATERIALS AND METHODS

### 2.1 Study site

**FIGURE 1:**
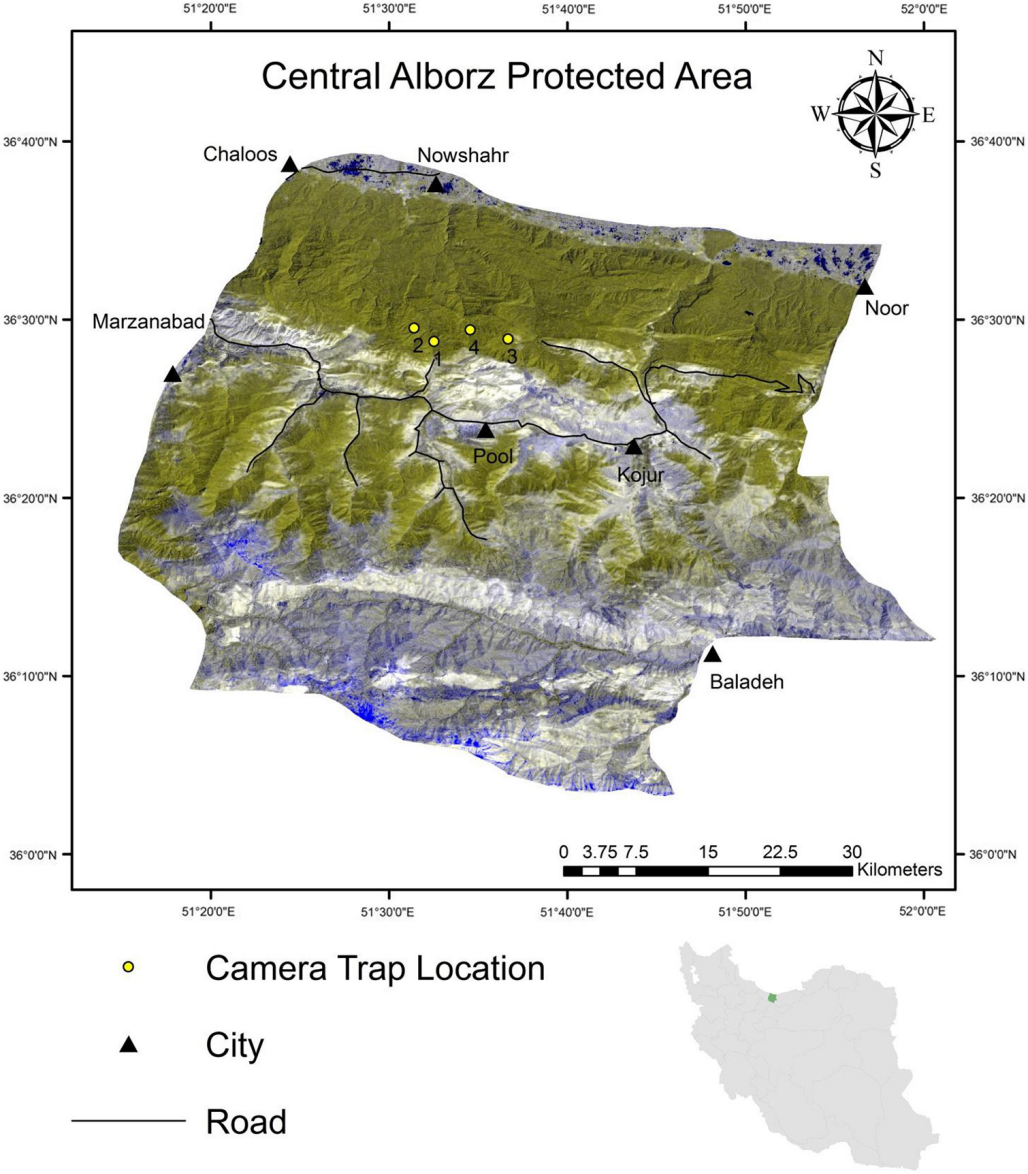
Location of camera trap stations (each at a mineral licking sites) during May–July 2019 in central Alborz protected area (CAPA), northern Iran

Our study was carried out in the Hyrcanian forests of Central Alborz Protected Area (hereafter CAPA; 36°29′N and 51°34′E), Mazandaran, northern Iran. Also known as a UNESCO World Heritage, our field sampling was done in Kojour area along the northern slopes of the CAPA, at an altitude of 1500–2000 m above sea level (Figure 1). The average annual rainfall and temperature are 1300 mm and 16.1°C, respectively (Rahimi et al., 2013). The study area has dense forest cover and protected animal species, such as Persian leopard (*Panthera pardus tulliana*), brown bear (*Ursus arctos*), and gray wolf (*Canis lupus*) (Nezami & Farhadinia, 2011; Salmanpour et al., 2021). According to Mazandaran Bureau of the Department of Environment (Unpublished data), it is guessed that ca. 300 Caspian red deers, 250–400 roe deers, and 600–800 wild pigs exist in the area in 2020.

### 2.2 Data collection

Based on the local ecological knowledge from hunters and rangers, we identified four naturally occurring mineral licks used by herbivores (Figure 1). Identified sites were at a mean spacing of 4.6 (SD 2.1) km. We then equipped all fours sites with digital camera traps (Trail Camera DH‐8) with infrared flash, each working for 40 nights between May and July 2019, overlapping with the lactation period of herbivores in northern Iran (Ziaie, 2008) when the need to mineral nutrients peaks, particularly sodium (Ceacero et al., 2009). Longer survey period was not possible because of the high rate of vandalism. All camera traps were set similarly at 40 cm from the ground, under the shade of trees and northward to avoid direct sunshine into the camera lens. We did not use any lure to attract the animals. They were set to video mode for 1 min, with 30 s delay between consecutive videos and were inspected every 2–3 weeks to ensure their functionality and to save the videos recorded on the memory card using a portable device.

We also obtained water sample from each mineral lick. Water samples were stored at a temperature of +5°C and away from sunlight. In the lab, samples were examined for the concentration of high‐consumption elements, such as sodium, potassium, magnesium, and trace elements, such as iron, zinc, manganese, copper, cobalt as well as their pH. We used Atomic Absorption Spectroscopy (AAS) by SPECTR AA‐20 to chemically analyze and measure the amount of elements (Wright & Stuczynski, 1996) in mineral water. We also determined the pH of the mineral licks using Jenway 3505 pH meter.

### 2.3 Statistical analysis

All statistical analyses were performed in R 4.0.2 (R Core Team, 2020). First, following Nouvellet et al. (2012), we converted detection times to sun time using the “sunTime” function in the R package overlap (Meredith & Ridout, 2020), using sunrise and sunset data from the National Oceanic & Atmospheric Administration.

We used the nonparametric kernel density estimation method for circular data (Ridout & Linkie, 2009) using the R package overlap (Meredith & Ridout, 2020). After converting time to radians, a probability density curve was produced for each species and the degree of interspecific temporal overlap was then estimated with the coefficient of overlapping, ∆, where a value of 0 represents no overlap and 1 represents complete overlap. We used the ∆1 variant because the number of photographic detections was <50 (Meredith & Ridout, 2020). Following recommendations in Meredith and Ridout (2020), a smoothing parameter of 0.8 was applied to overlap estimates using ∆1. We calculated 95% confidence intervals for the ∆1 from 10,000 smoothed bootstrap samples. Finally, to evaluate whether pairs of species had similar activity patterns, we used Watson's two‐sample *U* ^2^ test using the “circular” package in R (Lund et al., 2017) which tests two independent samples for common distribution.

We followed Azevedo et al. (2018) to categorize the temporal pattern of deer visitations to the licking sites. Accordingly, we defined them as either diurnal (<10% detections at night), mostly diurnal (10%–29% detections at night), nocturnal (≥90% detections at night), mostly nocturnal (70%–89% detections at night), or crepuscular (50% detections during dawn or dusk), with any falling outside of these categories classed as cathemeral.

We then used Generalized Additive Models using the “mgcv” package with Poisson distribution (Wood & Wood, 2015) to evaluate the relationship between the average ambient temperature and the visitation rate, the latter defined as the number of independent detections per day summed across the four camera trap stations. The residuals were checked against the predicted values using “DHARMa” package (Hartig & Hartig, 2017). Multiple detections at the same camera trap station <30 min apart were removed from the dataset to ensure the independence of detections (Rouse et al., 2021).

We then computed Spearman's rank correlation test to assess the relationship of the visitation rates among the four camera traps stations. The same test was run to evaluate the association between the ambient temperature and the visitation rate. Also, we used linear models to investigate the association between the visitation rates of deer species and the concentrations of sodium and potassium, which are both intensively used by deer (Ceacero et al., 2009).

The average ambient temperature was obtained from the temperature stamped on photos. We averaged the temperatures recorded on images obtained between 1000 and 1100 every day among the four camera traps stations. This period overlapped with high avian activities at the licking sites to find water, which resulted in triggering the camera traps and recording the temperature based on the camera trap inner thermometer. All camera traps were set similarly in terms of height and direction. We therefore assumed that the obtained ambient temperatures are comparable across the camera traps. Species recorded fewer than 10 times during the study period were excluded from analyses due to lack of data.

## 3 RESULTS

During 160 trap nights evenly distributed among four licking sites, a total of 53 independent mineral lick visits were obtained, exclusively belonged to three species of herbivores, Caspian red deer (*n* = 21), roe deer (*n* = 26), and wild pig (*n* = 6), the latter was excluded from our analysis because of the small number of detections (Table 1). We never detected multiple individuals or species in a visit. We also never detected any Caspian red deer calves at the licking sites. However, we observed variable lick visitations among sex classes. The sex ratio of visiting Caspian red deer was highly skewed toward females (3M:18F), whereas it was more balanced among visiting roe deer (11M:15F). The visitation rates did not show any evidence of pairwise correlation between camera trap stations for either deer species, except between camera trap stations 2 and 4 for roe deer (*r* = 0.34, *p* = .03).

**TABLE 1:** Visitation rates of large herbivores at four different mineral licks during May–July 2019 in central Alborz protected area, northern Iran

Mineral site number	Caspian red deer (*Cervus elaphus*)	Roe deer (*Capreolus capreolus*)	Wild pig (*Sus scrofa*)
1	0.13 (5)	0.20 (8)	0.03 (1)
2	0.08 (3)	0.15 (6)	0.05 (2)
3	0.20 (8)	0.18 (7)	0.00 (0)
4	0.13 (5)	0.13 (5)	0.08 (3)

*Note*: Visitation rates are expressed as the number of visits per 40 day and the number of independent events are given in parentheses.

Importantly, although present in the area based on sporadic camera trapping efforts along trails and systematic surveys, no large carnivores, such as gray wolf, Persian leopard, and brown bear, were detected at mineral licking sites. At two mineral licking sites, we found recently built poaching hides, which were both destroyed. However, we never detected any human on photos during the sampling, including poachers.

**FIGURE 2:**
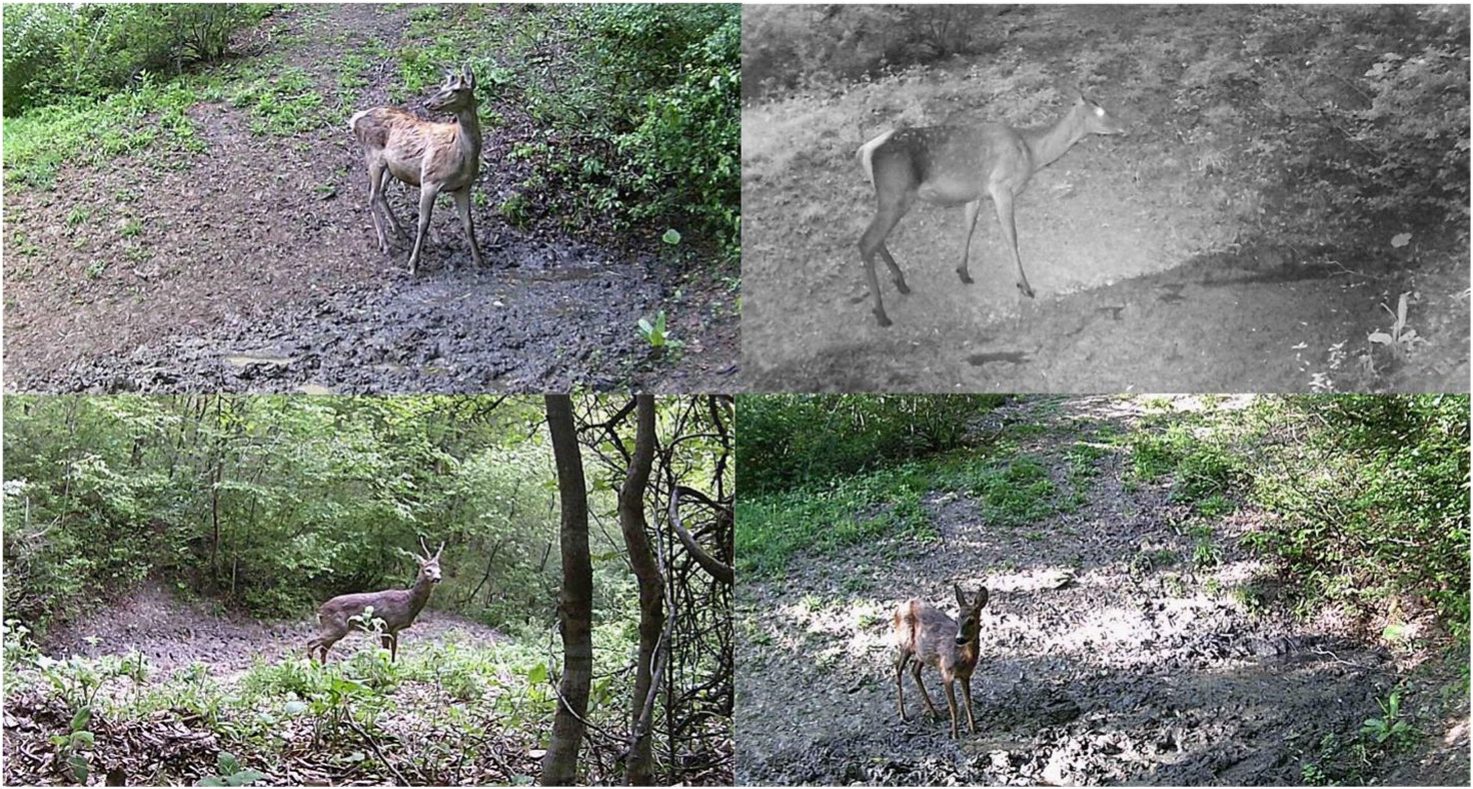
Some examples of Caspian red deer and roe deer at mineral licking sites in the central Alborz protected area (CAPA), northern Iran

**FIGURE 3:**
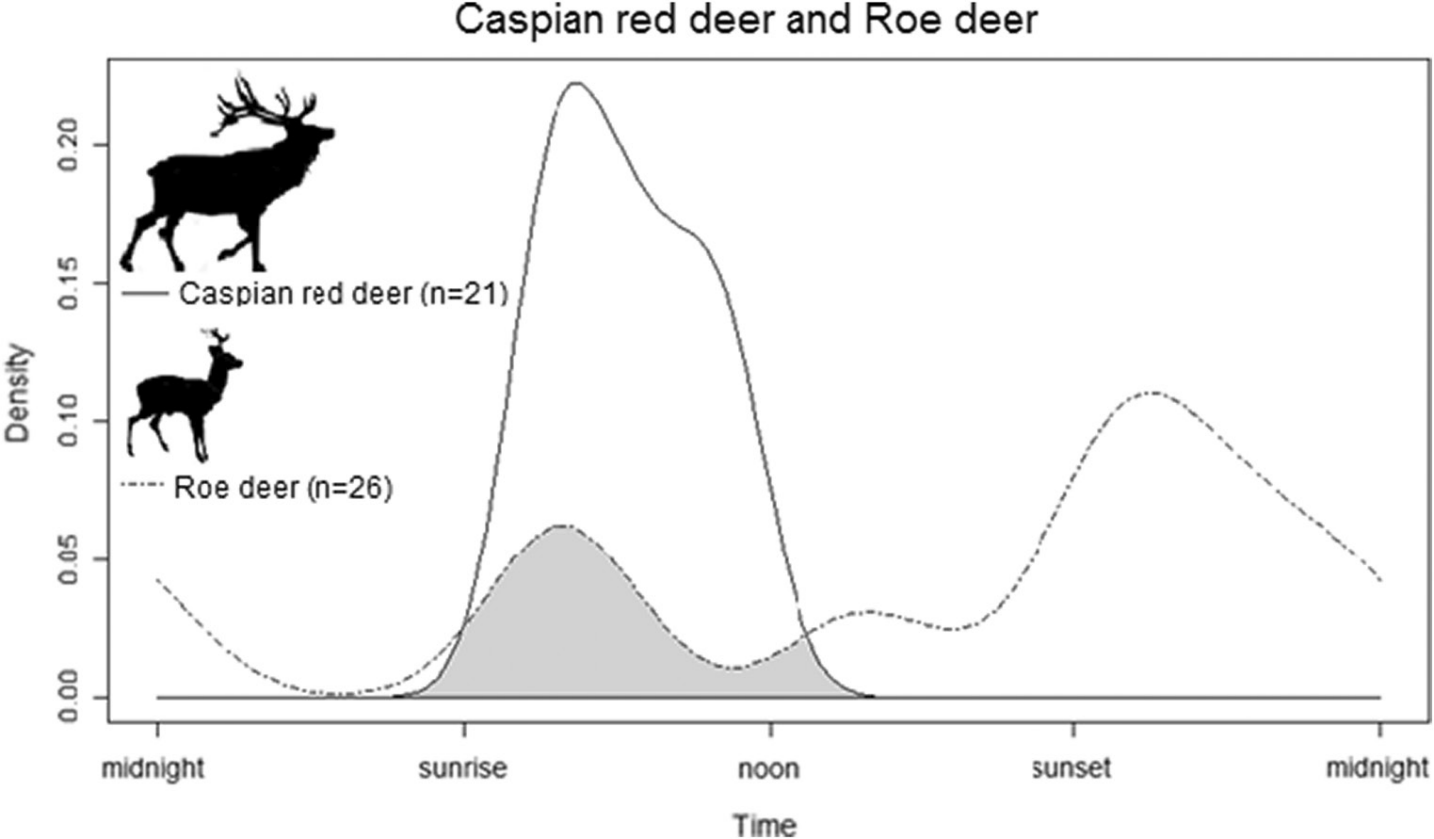
Temporal overlap plot of Caspian red deer (solid line) and roe deer (dashed line; Δ = 0.25). Gray areas underneath density curves represent the overlap coefficient, Δ

The activity patterns of Caspian red deer tended to be unimodal during visitations to the licking sites, exclusively occurring between 05:00 and 10:00, whereas roe deer visits followed a bimodal pattern, with peaks of activity after sunrise and sunset, including 23.1% occurring during nocturnal hours (20:40–04.10; Figure 2). Following circadian activity categories defined by Azevedo et al. (2018), the species‐level distribution of detections corresponded to diurnal for red deer and cathemeral for roe deer activity pattern. The coefficient of overlapping indicated a low degree of overlap (∆_1_ = 0.25 [CI = 0.01–0.39]) between Caspian red deer and roe deer (Figure 3), but without any evidence of different activity curves (*U* ^2^ = 0.62, *p* = .19).

**FIGURE 4:**
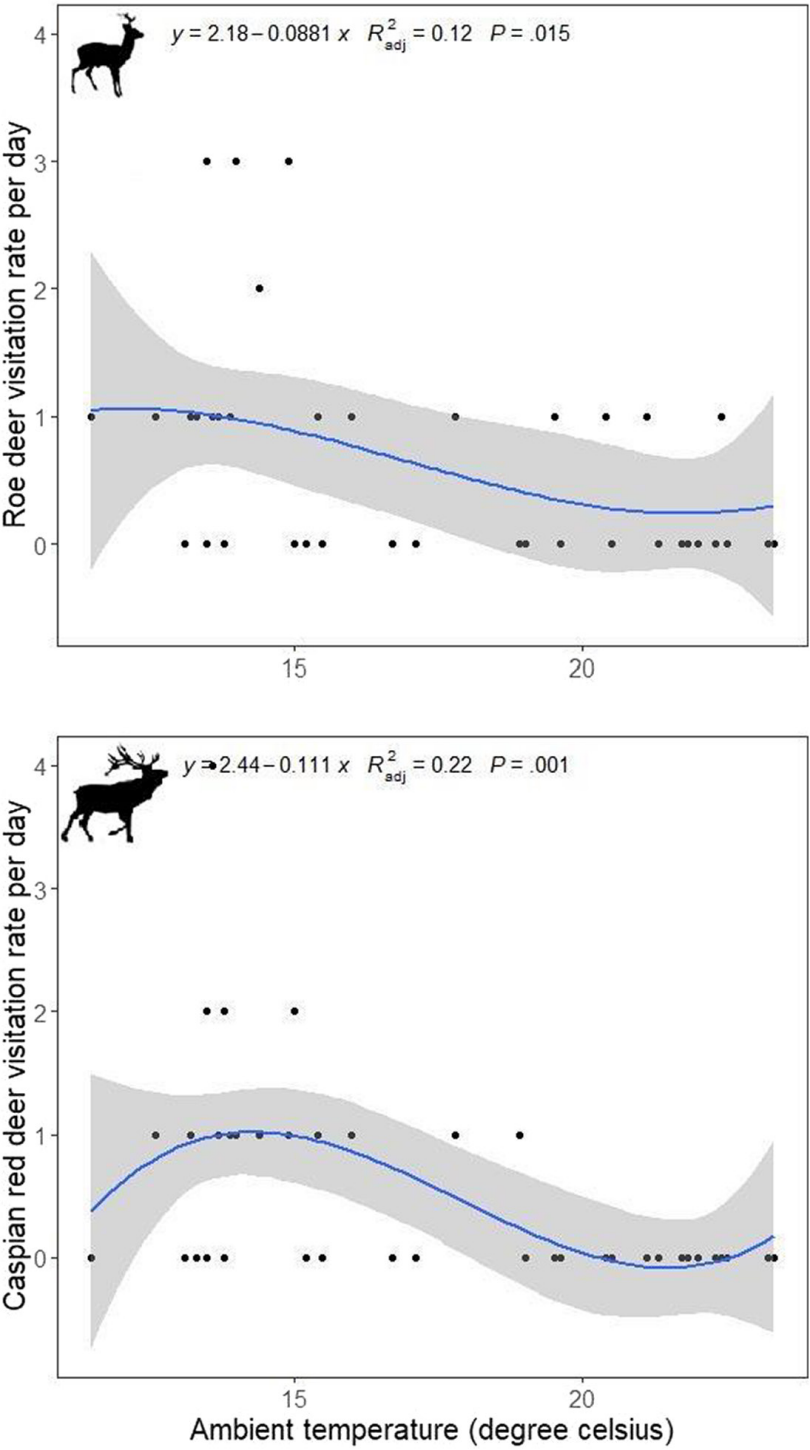
The association between average ambient temperature and the visitation rate of deer species by camera traps in in central Alborz protected area, northern Iran based on generalized linear model. The shaded area denotes confidence interval 95%.

There was a negative nonlinear relationship between the average ambient temperature and the visitation rates of deer species by camera traps. Accordingly, higher average ambient temperatures were associated with lower visitation rates at the licking sites by Caspian red deer (*β* = −2.85 ± SE 0.51; *p* < .01) and roe deer (*β* = −1.91 ± SE 0.22; *p* < .01, Figure 4). Our Spearman's rank correlation also showed that there is evidence of negative association between the ambient temperature and the visitation rate for Caspian red deer (*r* = −0.30, *p* < .05). However, the evidence for the relationship of roe deer visitations and ambient temperature was marginally significant (*r* = −0.14, *p* = .08). We also found that chemical elements were present in licking sites at different concentrations, showing that forest mineral licks can be sources of several elements, such as potassium, magnesium, iron, copper and cobalt. Importantly, three licking sites had high concentration of sodium (Table 2). Also, all licking sites were in the near‐neutral range in terms of the pH. There was no evidence for association between the concentration of sodium and either of deer visitation rates (Caspian red deer: *F*_1,2_ = 3.53, *p* = .20; roe deer: *F*_1,2_ = 0.01, *p* = .93). Similarly, we did not find any evidence supporting that the visitation rate is related to potassium concentration (Caspian red deer: *F*_1,2_ = 0.35, *p* = .65; roe deer: *F*_1,2_ = 0.47, *p* = .61).

**TABLE 2:** The concentration of chemical elements assessed in mineral licks in central Alborz protected area, northern Iran

Mineral site number	Na (ppm)	K (ppm)	Mg (ppm)	Fe (ppm)	Zn (ppm)	Mn (ppm)	Cu (ppm)	Co (ppm)	pH
1	97.19	7.45	11.81	0.72	0	0	0.06	0.01	7.44
2	6.97	2.08	8.47	0.99	0	0	0.03	0.00	7.10
3	129.45	9.02	5.36	2.01	0	0	0.09	0.05	6.80
4	127.66	16.47	7.82	0.32	0	0	0.03	0.01	6.89

## 4 DISCUSSION

Our study showed the exclusive use of mineral licks by herbivores during the survey period in northern Iran, particularly deer species. Deer species are often exposed to deficiency in different elements, particularly sodium in their diet (Ceacero et al., 2009). Therefore, mineral licking sites are vital resources for deer to maintain their homeostasis and natural metabolism. Despite the occurrence of carnivores such as brown bear, Persian leopard, and stone marten (*Martes foina*) in CAPA (Farhadinia & Valizadegan, 2015; Salmanpour et al., 2021), the exclusive use of mineral licks by herbivores during the survey period was in contrary to other studies from tropical forests where non‐herbivore species also visited the licking sites (Griffiths et al., 2020; Matsubayashi et al., 2006; Razali et al., 2020).

Higher ambient temperature was negatively associated with visitation rate in both deer species, which can be explained by multiple factors, such as migration to higher and colder altitudes, increased occurrence of insects around licking sites and lower need to minerals in association with reduced lactation. Alternatively, the lower visitation rate in higher temperature can be also explained by a reduction in activity levels of deer which is an adaptive strategy to avoid heat stresses in herbivores (Brivio et al., 2016; Grignolio et al., 2018; Zanni et al., 2021). Nonetheless, small sample sizes and a lack of replication across multiple years require us to view our findings as suggestive rather than conclusive, and to concede that further research is necessary to evaluate the spatiotemporal interaction of these herbivores and how mineral licking behavior varies with different life stages such as lactation or antlerogenesis in northern Iran. Also, future studies are recommended to investigate the effects of other covariates such as habitat type and human disturbance on the visitation rate of the endangered herbivores in this area.

The concentration of minerals varied remarkably between the licking sites. For example, the concentration of sodium ranged between 97 and 129 ppm with one licking site as low as 7 ppm. However, there was no evidence for any association between the mineral concentration and the visitation rates by each deer species. There are two explanations. First, deer may suffer from high deficiency of sodium, so even relatively low concentrations is crucial for them. Second, other elements are needed in the diet of deer which attracts them to these mineral licks. Absence of calves at mineral licking sites is in accordance with the low concentration of cobalt in studied mineral licks, an element mainly needed by calves as a requirement for growing ruminants which is are about 10 times higher than for adults (Ceacero et al., 2010). In contrast, during lactation (our study period), increased water intake and increased potassium concentration in plant tissues increase the need for sodium in adult females (Kaspari, 2020). Given that we found recently built poaching hides at two mineral licking sites, high risk of poaching associated with these sites can also explain the absence of calves at licking sites.

We also found that herbivores are attracted to the artificial salt sources provided by cattle ranchers for their livestock, based on personal observations made by MK, RE, and SGh, who work as ranger in the CAPA. These artificial mineral licks can cause two problems for deer. First, they can increase the likelihood of pathogen transmission from domestic to wild herbivores (Plummer et al., 2018). Second, higher detectability around mineral licks makes deer species prone to poachers. Interviews with elder hunters in the study area indicated occasional use of mineral licks as a bait to lure deer species for hunting in CAPA.

This research shed light on the importance of geological features as a valuable resource to Caspian red deer and roe deer in the form of electrolyte supplementation for proper biological functioning. In addition to further investigation on the ecological functions of licking sites, it is also necessary to identify and protect these resources and to include them as a key habitat requirement in spatial planning for the species. Similarly, it is recommended that local cattle herders are informed about the potential risk of disease transmission between domestic and wild herbivores.

## AUTHOR CONTRIBUTIONS

**Farid Salmanpour:** Data curation (equal); formal analysis (equal); funding acquisition (equal); investigation (equal); project administration (equal); writing – original draft (equal). **Zahra Shakoori:** Data curation (equal); investigation (equal); writing – review and editing (equal). **Mehdi Kia:** Data collection (equal). **Rahman Eshaghi:** Data collection (equal). **Mehdi Ghaderi:** Data collection (equal). **Saied Ghomi:** Data collection (equal). **Reza Kaveh:** Data collection (equal). **Kuros Rabie:** Data collection (equal). **Bahram H. Kiabi:** Supervision (equal). **Mohammad S. Farhadinia:** Software (equal); writing – review and editing (equal).

## FUNDING INFORMATION

This research did not receive any specific grant from funding agencies in the public, commercial, or not‐for‐profit sectors.

### OPEN RESEARCH BADGES

This article has earned Open Data and Open Materials badges. Data and materials are available at https://figshare.com/s/e5686a13ba3235fbf1bc.

## Supporting information


Appendix S1

## Data Availability

Datasets analyzed during the current study are available on Figshare as https://figshare.com/s/e5686a13ba3235fbf1bc.
