# Supplementary material for: Mineral lick use by a community of large herbivores in northern Iran
Source: Ecol Evol. 2023 Jan 18;13(1):e9731. doi: 10.1002/ece3.9731 (PMC9849147; doi:10.1002/ece3.9731)
Supplement: Supplementary file 1 — Appendix S1 [file ECE3-13-e9731-s001.docx]

Dear Editors

In the Authors & Institutions section to include our authors in the third place from Mazandaran Provincial Office, Department of Environment, Mazandaran, Iran was not available in the default options of the site, all 6 of them collaborated in the field studies field. Please consider the authors of this article in the following order:

1- Farid Salmanpour^*^

Department of Biodiversity and Ecosystem Management, Research Institute of Environmental Sciences, Shahid Beheshti University, Tehran, Iran, Email: [farid.salmanpour@yahoo.com](mailto:farid.salmanpour@yahoo.com)

2- Zahra Shakoori

Department of Plant Science and Biotechnology, Faculty of Science and Biotechnology, Shahid Beheshti University, Tehran, Iran Email: [zahrashakoori1992@yahoo.com](mailto:zahrashakoori1992@yahoo.com)

3- Mehdi Kia

Mazandaran Provincial Office, Department of Environment, Mazandaran, Iran Email: [mkia608@gmail.com](mailto:mkia608@gmail.com)

3- Rahman Eshaghi

Mazandaran Provincial Office, Department of Environment, Mazandaran, Iran Email: [rahmaneshaghi63@yahoo.com](mailto:rahmaneshaghi63@yahoo.com)

3- Mehdi Ghaderi

Mazandaran Provincial Office, Department of Environment, Mazandaran, Iran Email: [mehdiporia5@gmail.com](mailto:mehdiporia5@gmail.com)

3- Saied Ghomi

Mazandaran Provincial Office, Department of Environment, Mazandaran, Iran Email: [saeedghomi69@gmail.com](mailto:saeedghomi69@gmail.com)

3- Reza Kaveh

Mazandaran Provincial Office, Department of Environment, Mazandaran, Iran Email: [kavereza9@gmail.com](mailto:kavereza9@gmail.com)

3- Kuros Rabie

Mazandaran Provincial Office, Department of Environment, Mazandaran, Iran Email: [korosrabii@yahoo.com](mailto:korosrabii@yahoo.com)

4- Bahram H. Kiabi

Department of Animal Sciences and Marine Biology, Faculty of Life Sciences and Biotechnology, Shahid Beheshti University, Tehran, Iran, Email: [b.h.kiabi@gmail.com](mailto:b.h.kiabi@gmail.com)

5- Mohammad S. Farhadinia

Oxford Martin School and Department of Zoology, University of Oxford, UK, Email: [mohammad.farhadinia@zoo.ox.ac.uk](mailto:mohammad.farhadinia@zoo.ox.ac.uk)

Thanks and best wishes

Farid Salmanpour
